# Novel In Vivo Assessment of Antimicrobial Efficacy of Ciprofloxacin Loaded Mesoporous Silica Nanoparticles against *Salmonella typhimurium* Infection

**DOI:** 10.3390/ph15030357

**Published:** 2022-03-15

**Authors:** Maher N. Alandiyjany, Ahmed S. Abdelaziz, Ahmed Abdelfattah-Hassan, Wael A. H. Hegazy, Arwa A. Hassan, Sara T. Elazab, Eman A. A. Mohamed, Eman S. El-Shetry, Ayman A. Saleh, Naser A. ElSawy, Doaa Ibrahim

**Affiliations:** 1 Laboratory Medicine Department, Faculty of Applied Medical Sciences, Umm Al-Qura University, Makkah 21955, Saudi Arabia; mnandiyjany@uqu.edu.sa; 2 Quality and Development Affair, Batterjee Medical College, Jeddah 21442, Saudi Arabia; 3 Department of Pharmacology, Faculty of Veterinary Medicine, Zagazig University, Zagazig 44519, Egypt; asabdelaziz@vet.zu.edu.eg; 4 Department of Anatomy and Embryology, Faculty of Veterinary Medicine, Zagazig University, Zagazig 44511, Egypt; aabdelfattah@vet.zu.edu.eg; 5 Biomedical Sciences Program, University of Science and Technology, Zewail City of Science and Technology, October Gardens, 6th of October, Giza 12578, Egypt; 6 Department of Microbiology and Immunology, Faculty of Pharmacy, Zagazig University, Zagazig 44511, Egypt; waelmhegazy@daad-alumni.de; 7 Department of Pharmacology & Toxicology, Faculty of Pharmacy & Pharmaceutical Industries, Sinai University, El-Arish 45511, Egypt; arwa.ahmed@su.edu.eg; 8 Department of Pharmacology, Faculty of Veterinary Medicine, Mansoura University, Mansoura 35516, Egypt; sarataha1@mans.edu.eg; 9 Department of Microbiology, Faculty of Veterinary Medicine, Zagazig University, Zagazig 44519, Egypt; eman.zewail@hotmail.com; 10 Department of Human Anatomy and Embryology, Faculty of Medicine, Zagazig University, Zagazig 44511, Egypt; emanelshetry@zu.edu.eg; 11 Department of Animal Wealth Development, Veterinary Genetics & Genetic Engineering, Faculty of Veterinary Medicine, Zagazig University, Zagazig 44519, Egypt; lateefsaleh@yahoo.com; 12 Department of Anatomy & Embryology, Faculty of Medicine, Zagazig University, Zagazig 44511, Egypt; naser_elsawy@ymail.com; 13 Department of Nutrition and Clinical Nutrition, Faculty of Veterinary Medicine, Zagazig University, Zagazig 44511, Egypt

**Keywords:** *Salmonella typhimurium*, ciprofloxacin, drug-loaded nanoparticles, qRT-PCR, histopathological examination

## Abstract

*Salmonella enterica* serovar Typhimurium (*S. typhimurium*) is known for its intracellular survival, evading the robust inflammation and adaptive immune response of the host. The emergence of decreased ciprofloxacin (CIP) susceptibility (DCS) requires a prolonged antibiotic course with increased dosage, leading to threatening, adverse effects. Moreover, antibiotic-resistant bacteria can persist in biofilms, causing serious diseases. Hence, we validated the in vitro and in vivo efficacy of ciprofloxacin-loaded mesoporous silica nanoparticles (CIP–MSN) using a rat model of salmonella infection to compare the oral efficacy of 5 mg/kg body weight CIP–MSN and a traditional treatment regimen with 10 mg/kg CIP postinfection. Our results revealed that mesoporous silica particles can regulate the release rate of CIP with an MIC of 0.03125 mg/L against DCS *S. typhimurium* with a greater than 50% reduction of biofilm formation without significantly affecting the viable cells residing within the biofilm, and a sub-inhibitory concentration of CIP–MSN significantly reduced *inv*A and *Fim*A gene expressions. Furthermore, oral supplementation of CIP–MSN had an insignificant effect on all blood parameter values as well as on liver and kidney function parameters. MPO and NO activities that are key mediators of oxidative stress were abolished by CIP–MSN supplementation. Additionally, CIP–MSN supplementation has a promising role in attenuating the elevated secretion of pro-inflammatory cytokines and chemokines in serum from *S. typhimurium*-infected rats with a reduction in pro-apoptotic gene expression, resulting in reduced *S. typhimurium*-induced hepatic apoptosis. This counteracted the negative effects of the *S. typhimurium* challenge, as seen in a corrected histopathological picture of both the intestine and liver, along with increased bacterial clearance. We concluded that, compared with a normal ciprofloxacin treatment regime, MSN particles loaded with a half-dose of ciprofloxacin exhibited controlled release of the antibiotic, which can prolong the antibacterial effect.

## 1. Introduction

*Salmonella enterica* serovar Typhimurium is a Gram-negative member of the family Enterobacteriaceae and is considered the second-most common cause of food poisoning associated with the consumption of contaminated food or water. It causes gastroenteritis, typhoid, and paratyphoid diseases in humans, and it can infect a wide range of hosts, including reptiles, birds, and mammals [1]. It is known that this bacterial pathogen is capable of intracellular survival by replicating inside the host cell in specialized vacuoles called *Salmonella*-containing vacuoles (SCVs). This clever mechanism causes persistent infection by allowing evasion of the robust inflammation and adaptive immune response of the host [2]. In addition, *Salmonella* can invade and translocate across the gut–epithelial barrier and infect the phagocytes, gaining access to the lymphatics and bloodstream, which allows the bacteria to spread to the liver and spleen [3]. Conventional antibacterial regimens have difficulty treating salmonella infection because of the intracellular survival and defenses of the bacteria [4]. Moreover, the increased prevalence of antimicrobial resistance limits the use of traditional antimicrobial agents, such as ampicillin, chloramphenicol, and trimethoprim-sulfonamide combinations, leading to the emergence of antimicrobial resistance among bacterial strains isolated from humans [5]. Fluoroquinolones, especially ciprofloxacin, have become the alternative option for treating salmonella infections [6]. However, the recent emergence of decreased ciprofloxacin susceptibility (DCS) or even resistance has led to treatment failures [7]. Delivering free antibiotics intracellularly has several limitations as a prolonged antibiotic course of 7 d, and the increased dosage required for treating salmonella infection leads to threatening, adverse effects [8]. Additionally, the WHO described a growing concern about *Salmonella’s* ability to form biofilms of fimbriae components [9], along with alarming antimicrobial resistance that presents a major threat to human and veterinary medicine [10].

These limitations have highlighted the importance of developing a biocompatible drug-delivery system with a high-loading capacity that controls drug release, allowing reduced dosage without compromising efficacy in treating intracellular pathogens [11]. Improvement of the overall pharmacokinetics, reduction of antimicrobial resistance, enhancement of the solubility of some antibiotics, and a wider therapeutic index are some benefits of developing drug-delivery systems [12]. Mesoporous silica nanoparticles show great promise as a biomedical application [13]. Their fabrication method at low temperatures enables them to carry biologically active agents as a drug-delivery system [14]. In addition, their unique properties include good biocompatibility, low hemolytic effect, and low toxicity. The high surface area and highly permeable, porous shell enable high drug-loading capacity and delayed release of antibacterial agents, which reduce the frequency of the dosages [15]. As shown in many previous reports, mesoporous silica nanoparticles (MSNs) as antibiotic delivery systems have been proven to boost the antimicrobial efficacy and safety profile of ciprofloxacin [16,17]. However, these reported studies were either conducted to evaluate in vitro antibacterial and cytotoxicity activity [17], or were in vivo survival assays prior to a murine oral *Salmonella typhimurium* infection model [16].

The above-mentioned studies provided a new sense of hope to further validate the in vitro efficacy of ciprofloxacin-loaded mesoporous silica nanoparticles against biofilm formation and the fold change in the mRNA expression of *iva*A and *Fim*A genes. Furthermore, the in vivo efficacy of a reduced dose of ciprofloxacin-loaded mesoporous silica nanoparticles compared with a normal ciprofloxacin treatment regime showed good biocompatibility and lower cytotoxicity after measuring the hematological profile and the liver and kidney function parameters. Interaction between the immune system and the ciprofloxacin-loaded mesoporous silica nanoparticles was evaluated through the antioxidant profile, pro-inflammatory cytokines, chemokines, and apoptosis regulator genes. Additionally, pathological changes and the bacterial clearance effect were evaluated.

## 2. Results

### 2.1. Characterization of Mesoporous Silica Nanoparticles (MSNs), Ciprofloxacin Loading, and Release

**Figure 1:**
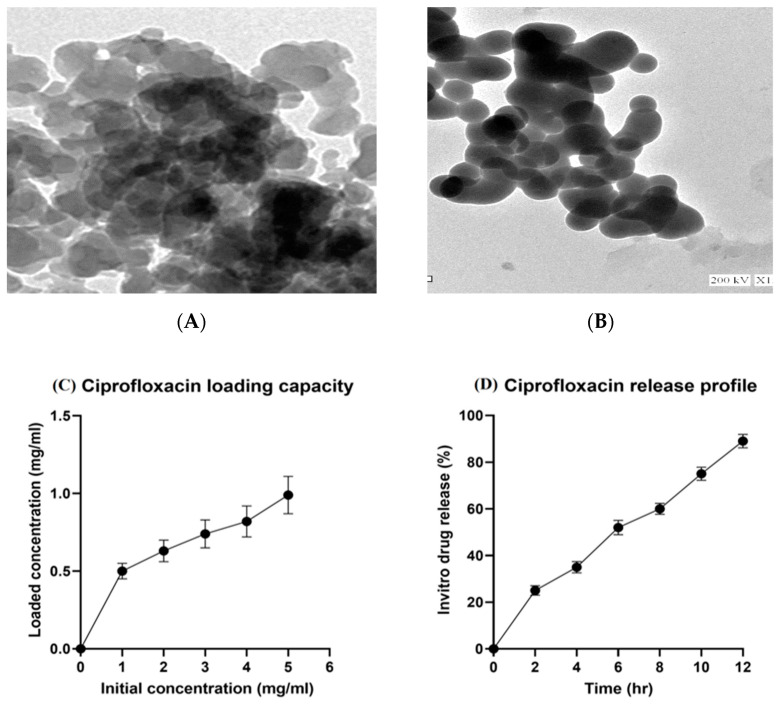
(**A,B**) Characterization of CIP–MSNs NPs by transmission electron microscopy (TEM); (**C**) Ciprofloxacin loading amount in relation to initial drug concentration (mg/mL); (**D**) percentage of ciprofloxacin in vitro release from CIP–MSNs over 12 h.

Characterization of mesoporous silica nanoparticles with ciprofloxacin (CIP–MSN) was carried out through transmission electron microscopy (TEM) (Figure 1A,B) at the National Center for Radiation Research and Technology (NCRRT), Atomic Energy Authority, Egypt. The loaded ciprofloxacin amount was increased by increasing the initial 5 mg/mL concentration of ciprofloxacin until it reached the maximum loading capacity of 1 mg for every 5 mg of MSN (Figure 1C). The in vitro release of ciprofloxacin from the CIP–MSN was gradual over 12 h, beginning with a 25% release in the first 2 h, followed by a cumulative and ongoing release until nearly 90% of the drug was released at 12 h after incubation (Figure 1D).

### 2.2. In Vitro Antibacterial Activity of CIP–MSN against Decreased CIP Susceptible Salmonella typhimurium

The antibacterial activity of prepared CIP–MSN was tested against the *Salmonella typhimurium* strain with decreased CIP susceptibility and compared with that of MSN and CIP. The results showed a significant variation in the inhibition zone of CIP–MSNs, along with both ciprofloxacin and unloaded mesoporous silica nanoparticles. The maximum antibacterial activity was observed in CIP–MSN with 40.5 ± 0.4 mm diameter of inhibition zone compared with 3.4 ± 0.37 and 8.7 ± 0.2 mm diameter inhibition zones of MSN and CIP, respectively (Table 1). The *Salmonella typhimurium* strain with decreased CIP susceptibility had an MIC for ciprofloxacin of 1.0 mg/L, compared with CIP–MSN. The antibacterial efficacy was confirmed with an MIC of CIP–MSN of 0.03125 mg/L, while MSN exhibited no inhibitory effect on visible bacterial growth (Table 1). Furthermore, the MBC value was two-fold higher than MIC values, indicating their bactericidal effect.

### 2.3. Biofilm Inhibition and Transcriptional Modulatory Effect of CIP–MSN

**Figure 2:**
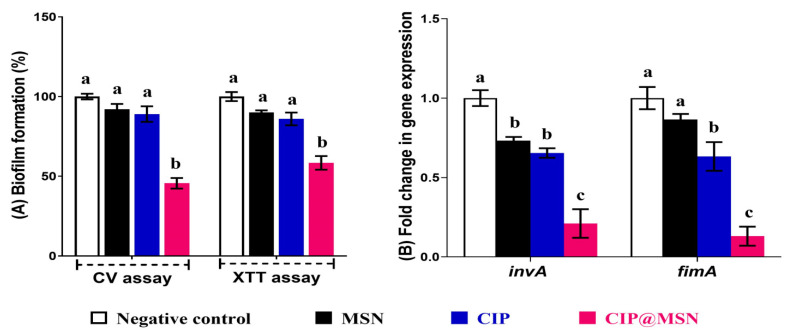
CIP–MSN reduces the biofilm formation of *S. typhimurium* compared with CIP alone, MSN, and NC(negative control; untreated bacteria). (**A**) Biofilm formation of tested *S. typhimurium* in the presence of CIP–MSN was detected by crystal violet staining and quantified by measuring the OD590, and cell viability within biofilm was detected by XTT assay and quantified by measuring the OD490; (**B**) Relative gene expression of *S. typhimurium* virulence genes *Fim*A and *inv*A upon treatment with CIP–MSN were calculated using the ΔΔCT method and expressed as fold change. 16S rRNA was used as the endogenous control. Each column shows the mean ± SD of three independent experiments. ^a–c^ Means within the same column carrying different superscripts are significantly different at *p* < 0.05.

Our results revealed that a sub-MIC concentration of CIP–MSN significantly reduced biofilm formation to 45% through a CV assay where 58% of cells remained viable upon performing an antibiofilm assay with the XTT method. This confirmed the effectiveness of a sub-MIC (1/2 MIC) concentration at inhibiting the extracellular polymeric substances (EPS) that form the biofilm matrix, with remaining viable cells residing within the biofilm. However, upon comparison to CIP and MSN, similar patterns were obtained with the CV and XTT assay, as it showed a nonsignificant reduction in biofilm (89 and 92%, respectively) (*p* > 0.05) (Figure 2A).

The modulatory effect of CIP–MSN on the invasion and major fimbrial subunit associated genes were investigated by reverse transcriptase expression qPCR of *inv*A and *Fim*A in biofilm *S. typhimurium* culture (Figure 2B). Both genes were downregulated as the expression levels of *inv*A and *Fim*A in CIP–MSN-treated *S. typhimurium* were 0.21-fold and 0.13-fold, respectively, which were more effective than CIP alone (0.6-fold change for *inv*A and *Fim*A genes). As expected, the MSN-treated *S. typhimurium* did not produce a detectable change in *inv*A and *Fim*A expression compared with untreated *S. typhimurium* (*p* > 0.05).

### 2.4. Hematological, Biochemical, Antioxidant, and Immunological Effect of CIP–MSN on Blood, and Serum Constituents

Red blood cells (RBCs), hemoglobin concentration (Hb), and packed cell volume (PCV) were significantly elevated in CIP–MSN compared with the untreated challenged group, with red blood cells (RBCs) and hemoglobin concentration (Hb) levels nonsignificantly different from NC (Table 2). Moreover, based on the biochemical analysis of the blood serum constituents, the challenging with *S. typhimurium* adversely affected the liver and kidney function parameters, as represented by significantly increased alanine aminotransferase (ALT), aspartate aminotransferase (AST), urea, and creatinine levels at both 7 and 14 d postchallenge, while serum AST, ALT, uric acid, and creatinine levels were significantly reduced in the CIP–MSN-treated group regardless of challenge at both 7 and 14 d postchallenge, and their levels were nonsignificantly different from NC (Table 2). Concerning oxidative stress mediators, a significant decrease (*p* < 0.05) in NO and MPO levels at both time intervals in the group supplemented with CIP–MSN, when compared with the treated and control groups’ MPO levels, was nonsignificantly different from the negative group at 14 d postchallenge (Table 2). The results of pro-inflammatory cytokine and chemokine analysis in the group challenged with *S. typhimurium* showed a significant increase in blood serum levels of CXCL10, CXCL11, IFN-γ, IL-6, TNF-α, and CRP at both time points (Table 2). Meanwhile, supplementation with CIP–MSN caused a significant reduction in blood serum levels of CXCL10, CXCL11, IFN-γ, IL-6, TNF-α, and CRP at 7 and 14 d when compared with the treated and control groups. No significant difference was found in CXCL11, IFN-γ, IL-6, and TNF-α serum levels in the CIP–MSN-treated groups relative to the unchallenged NC group (Table 2).

### 2.5. Inhibitory Effect of CIP–MSN on Hepatic Salmonella typhimurium Load

**Figure 3:**
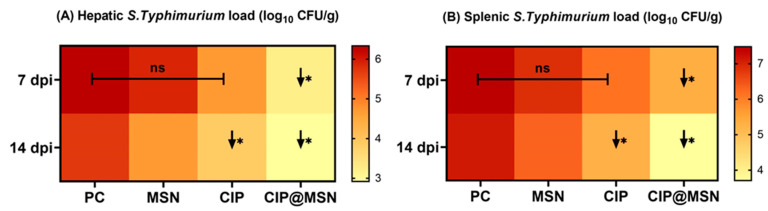
In vivo evaluation of CIP–MSN treatment on *S. typhimurium* bacterial load in (**A**) hepatic tissue and (**B**) splenic tissue at 7and 14 days post-infection (7 and 14 dpi) by real-time PCR quantification of DNA copies, and represented as log_10_ of the CFU per gram of tissue. PC (positive control): rats received a control diet without any addition and were orally challenged with *S. typhimurium*; MSN(mesoporous silica particles): rats received a control diet, were orally challenged with *S. typhimurium*, and received 10 mg/kg MSN orally twice a day for 3 days; CIP(ciprofloxacin): rats received a control diet, were orally challenged with *S. typhimurium*, and received 10 mg/kg CIP orally twice a day for 3 days; and CIP–MSN (MSN particles loaded with ciprofloxacin): rats received a control diet, were orally challenged with *S. typhimurium*, and received 5 mg/kg CIP orally twice a day for 3 days. Data are expressed as means ± SE (error bars). Arrows correspond to significant decrease (↓*) relative to the PC group (*p* < 0.05), and NS represents nonsignificant differences relative to the PC group (*p*-value > 0.05).

Quantitative, real-time PCR counting of *S. typhimurium* in the liver and spleen samples at 7 and 14 d postchallenge revealed significant lowering in the CIP–MSN-treated groups compared with the PC group (*p* < 0.05), and the bacterial load decreased steadily over time, whereas the reduction in log_10_ copies of *S. typhimurium* populations was evidenced by 3.2 and 2.9 log_10_ CFU/g at 7 and 14 d postinfection, respectively, in the liver sample, and 5.3 and 3.7 log_10_ CFU/g at 7 and 14 d postinfection, respectively, in the spleen sample (Figure 3). Changes in the log_10_ values of *S. typhimurium* CFU/g in MSN- and CIP-supplemented groups showed no statistically significant differences at 7 d postchallenge, and the bacterial load was significantly reduced in the CIP-treated group at 14 d postchallenge when compared to the PC group in both hepatic and splenic samples (Figure 3).

### 2.6. Pro-Inflammatory Cytokines Transcriptional Modulatory Effect of CIP–MSN

**Figure 4:**
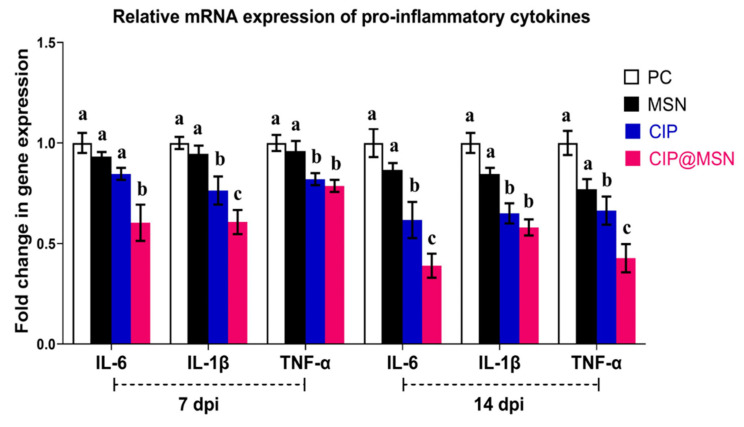
Relative mRNA expression levels of pro-inflammatory cytokines; interleukin-6 (IL-6), interleukin-β (IL-β) and tumor necrosis factor alpha (TNF-α) in the splenic tissue of rats treated with CIP–MSN compared with CIP alone at 7and 14days postinfection with *S. typhimurium* (7 and 14 dpi).The expression levels were calculated using the 2^−ΔΔCt^ method and expressed as fold change, and glyceraldehyde-3-phosphate dehydrogenase (GAPDH)was used as the endogenous control. PC (positive control): rats received a control diet without any addition and were orally challenged with *S. typhimurium*; MSN(mesoporous silica particles): rats received a control diet, were orally challenged with *S. typhimurium*, and received 10 mg/kg MSN orally twice a day for 3 days; CIP(ciprofloxacin): rats received a control diet, were orally challenged with *S. typhimurium*, and received 10 mg/kg CIP orally twice a day for 3 days; and CIP–MSN (MSN particles loaded with ciprofloxacin): rats received a control diet, were orally challenged with *S. typhimurium*, and received 5 mg/kg CIP orally twice a day for 3 days. Each column shows the mean ± SD of three independent experiments. ^a–c^ Means within the same column carrying different superscripts are significantly different at *p* < 0.05.

The mRNA expressions of pro-inflammatory cytokines interleukin-6 (IL-6), interleukin-1β (IL-1β), and tumor necrosis factor alpha (*TNF-α*) were examined at 7 and 14 d postinfection (Figure 4). The challenge with *S. typhimurium* adversely increased the mRNA expression of pro-inflammatory cytokines at both 7 and 14 d postchallenge (assigned a value of 1 arbitrary unit). The level of *IL-6* and *IL-1β* expression in the splenic tissue was significantly lower (*p* < 0.05) in the group that had received CIP–MSN, as compared with the other treatment and control groups; the most pronounced downregulation in the level of IL-6 (about a 0.3-fold reduction) was noticed at 14 d postinfection. Moreover, dietary supplementation of CIP–MSN decreased the transcriptional levels of TNF-α in a time-dependent manner compared to the PC group, as it reached about a 0.4-fold reduction by the end of the experiment (14 d postinfection).

### 2.7. Modulation of Pro-Apoptoticgenes Expression

**Figure 5:**
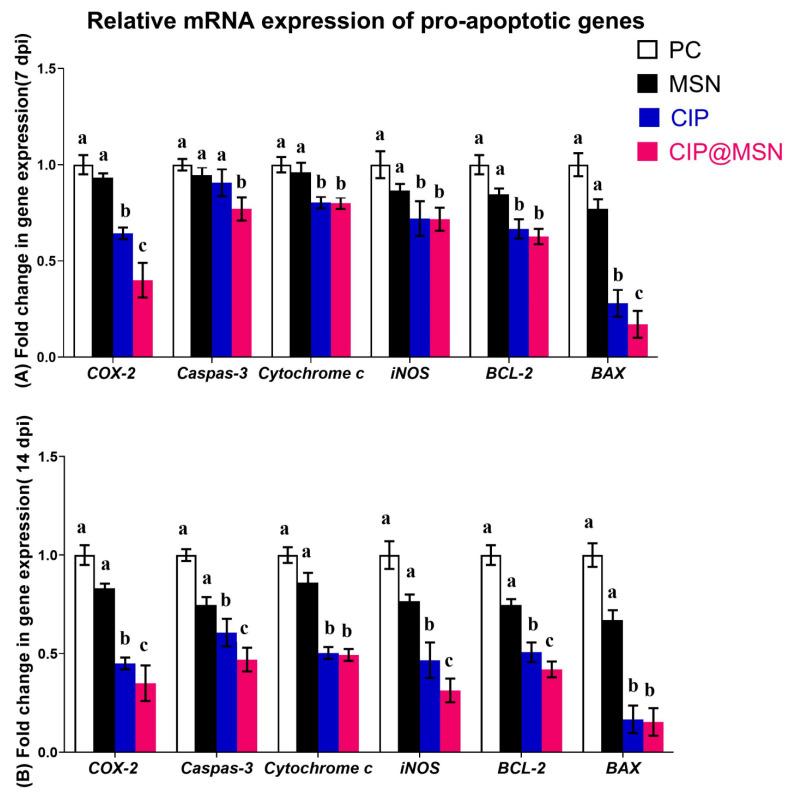
Relative mRNA expression levels of pro-apoptotic genes; Cyclooxygenase-2(*COX-2*), caspase-3, Cytochrome P450, inducible nitric oxide synthase (*iNOS*), B-cell lymphoma-2 (*Bcl-2*), and Bcl-2-associated X (*BAX*)in the splenic tissue of rats treated with CIP–MSN compared with CIP alone at (**A**) 7 and (**B**) 14 days postinfection with *S. typhimurium* (7 and 14 dpi).The expression levels were calculated using the -2^ΔΔCT^ method and expressed as fold change, and glyceraldehyde-3-phosphate dehydrogenase (GAPDH) was used as the endogenous control. PC (positive control): rats received a control diet without any addition and were orally challenged with *S. typhimurium*; MSN(mesoporous silica particles): rats received a control diet, were orally challenged with *S. typhimurium*, and received 10 mg/kg MSN orally twice a day for 3 days; CIP(ciprofloxacin): rats received a control diet, were orally challenged with *S. typhimurium*, and received 10 mg/kg CIP orally twice a day for 3 days; and CIP–MSN (MSN particles loaded with ciprofloxacin): rats received a control diet, were orally challenged with *S. typhimurium*, and received 5 mg/kg CIP orally twice a day for 3 days. Each column shows the mean ± SD of three independent experiments. ^a–c^ Means within the same column carrying different superscripts are significantly different at *p* < 0.05.

The expression levels of six pro-apoptotic genes (COX-2, caspase-3, P450, iNOS, Bcl-2, and BAX) of the CIP–MSN-treated and control groups at different time points (7 and 14 dpi) were investigated by qRT-PCR (Figure 5A,B).

Gene expression analysis found significant differences between the CIP–MSN-treated and control groups. Interestingly, we found that expression of COX-2 was greatly decreased in the CIP–MSN-treated group at 7 and 14 dpi, with 0.4- and 0.3-fold changes, respectively, compared with CIP treatment alone. CIP–MSN dietary supplementation downregulated the expression of caspase-3 significantly at both time intervals, with a cumulative effect (0.7- and 0.4-fold changes at 7 and 14 dpi, respectively). The CIP–MSN-treated group significantly downregulated expression of P450, and iNOS with no notable difference between the CIP–MSN- and CIP-treated groups at both time points. The lowest transcriptional expression of iNOS level was observed in the CIP–MSN-supplemented group at 14 dpi of about 0.3-fold when compared with the CIP-treated group and the PC group. Bcl-2 expression was significantly downregulated in the CIP–MSN-treated group compared with control groups, reaching a 0.4-fold decrease at 14 dpi. Meanwhile, the most downregulation occurred for the BAX gene, as observed in the CIP–MSN-supplemented group (about a 0.1-fold reduction) compared to the PC group at both time points.

### 2.8. Histopathological Evaluation

**Figure 6:**
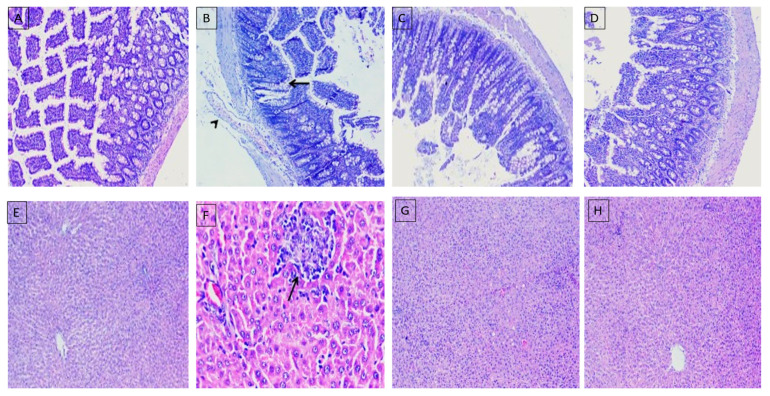
Representative photomicrographs using H&E staining of histological sections for intestines (**A**–**D**), and livers (**E**–**H**) of rats treated with CIP–MSN compared with CIP alone at 14 days postinfection with *S. typhimurium*. PC (positive control): rats received a control diet without any addition and were orally challenged with *S. typhimurium*; MSN(mesoporous silica particles): rats received a control diet, were orally challenged with *S. typhimurium*, and received 10 mg/kg MSN orally twice a day for 3 days; CIP(ciprofloxacin): rats received a control diet, were orally challenged with *S. typhimurium*, and received 10 mg/kg CIP orally twice a day for 3 days; and CIP–MSN (MSN particles loaded with ciprofloxacin): rats received a control diet, were orally challenged with *S. typhimurium*, and received 5 mg/kg CIP orally twice a day for 3 days.

Histopathological findings of intestinal and liver tissues post-*S. typhimurium* infection are presented in Figure 6. In the NC group, intestinal histomorphological structures showed normal mucosa, submucosa, musculosa, and serosa (Figure 6A). Meanwhile, in the PC group, the majority of intestinal sections showed enteritis, which was represented by dilated blood vessels, leukocytic infiltrations, metaplastic changes of the epithelial lining into goblet cells, and desquamated villous epithelium (Figure 6B). Additionally, the group treated with CIP alone displayed apparently normal intestinal layers (Figure 6C). However, goblet cell hyperplasia and denuded epithelium were detected in some examined sections. In the group treated with CIP–MSN, apparently normal mucosa, submucosa, and musculosa in most sections of intestine were detected (Figure 6D). Liver sections revealed normal cytoarchitectures of the hepatic cords, sinusoids, and stromal components in the NC group (Figure 6E). In the PC group, liver sections revealed unexpected multifocal necrotic areas that were mostly replaced by macrophages, and lymphocytes with severely dilated sinusoids accompanied by atrophied hepatic cords were seen within most examined sections (Figure 6F). In the group treated with CIP alone, liver sections showed some apparently normal hepatic parenchyma (Figure 6G), and in the group treated with CIP–MSN, preserved hepatic cords and hepatic vasculatures were more prominent in most liver tissues (Figure 6H).

## 3. Discussion

*Salmonella enterica* serovar Typhimurium is a common cause of persistent infection owing to its capacity for intracellular survival, biofilm formation, and evasion of the robust inflammatory response of the host, causing decreased ciprofloxacin susceptibility. Thus, an optimal strategy to treat these infections should deliver drugs with prolonged release from a single dose with good biocompatibility and lower toxicity. Previous reports demonstrating MSNs as antibiotic-delivery vehicles have been performed in vitro. There is a previous study reporting the in vivo survival in an assay of a murine oral *Salmonella typhimurium* infection model [16]. To the best of our knowledge, there are no previous reports validating in vitro antivirulence efficacy against biofilm formation; in vivo biosafety in blood parameters; and biochemical biomarkers of tissue injury or inflammation, pro-inflammatory cytokines and chemokines accompanied by pro-apoptotic gene expression, and histopathological effect.

Our results showed that the small particle sizes of the CIP-loaded MSN (CIP–MSN) play an important role in the internalization into cells, as reported previously, where submicron-sized particles can be taken up by M-cells and macrophages present in Peyer’s patches [19]. Accordingly, mesoporous silica particles can regulate the release rate of an antimicrobial agent [20]. Our in vitro antibacterial activity confirmed that the combination of mesoporous silica nanoparticles and CIP was responsible for higher antimicrobial activity when compared with the drug alone, and *Salmonella typhimurium* with decreased ciprofloxacin susceptibility (MIC 1.0 mg/L) was converted to 0.03125 mg/L. This indicated a reduction of antimicrobial resistance; the bacteria reverted to being susceptible to ciprofloxacin, according to the MIC break point of <0.125 mg/L. (EUCAST guidelines were applied for category interpretation for the different antibiotics [18] Silica nanoparticles alone showed no detrimental effects on bacteria [21].

The biofilm formation is a bacterial survival strategy leading to increased resistance to antibiotics [22]. Moreover, antibiotic-resistant bacteria can persist in biofilms and result in enhanced tolerance of adverse environmental conditions, causing serious infectious diseases [23]. To support the in vitro antibacterial enhancement effect of CIP–MSN, nanomaterials were proposed as an interventional strategy for the management of biofilm formation because of their high surface area-to-volume ratio and unique chemical and physical properties [24]. Our study reported a greater than 50% reduction in biofilm formation, with the remaining viable cells residing within the biofilm upon using a subinhibitory concentration of CIP–MSN, which was in accordance with another study that applied silver nanoparticle-doped nanoporous silica and reported a 70% reduction in biofilm survival [25].

*S. typhimurium* has been shown to produce a major fimbrial subunit and invasin A on the bacterial surface. Both of these vital virulence factors, encoded by the *Fim*A and *inv*A genes, play roles in mediating bacterial adherence to eukaryotic cells and facilitating the entry into intestinal epithelial cells, respectively, which are critical steps in successful colonization and pathogenesis [26]. Herein, the effect of CIP-loaded MSN on the reduction of biofilm formation was supported by significantly reduced *inv*A gene expressions and *Fim*A coding a major fimbrial subunit. This indicated the possible effect of this drug-delivery cargo on decreasing the *Salmonella* adherence and invasion of host cells and tissues [27,28].

The safety of the oral supplementation with a reduced dose of drug-delivery cargo was estimated through the evaluation of the hematological and biochemical parameters of rats. The pathophysiological status of the body related to infection and therapy was indicated by analysis of the hematological profile [29] and biochemical biomarkers for tissue injury or inflammation [30]. Herein, the administration of this drug-delivery cargo showed an insignificant effect on all blood parameter values and liver and kidney function parameters, indicating its good biocompatibility, low hemolytic activity, and lack of cytotoxicity in the liver and kidney function test. Consistent with a previous study [31], *Salmonella typhimurium* infection adversely affected the liver and kidney function parameters, resulting in a dramatic increase in ALT, AST, uric acid, and creatinine levels in serum, while these parameters were diminished with a reduced dose in CIP–MSN-treated groups, regardless of challenge, compared with the normal ciprofloxacin treatment regime.

The enhancing effects on the protective humoral immune response were indicated by detection of the key mediators of oxidative stress known to eliminate the invading bacterial pathogens, owing to the bactericidal action of MPO and NO produced from activated neutrophils and monocytes in the blood [32]. Our data revealed a significant elevation in oxidative stress mediators in response to *S. typhimurium* challenging, even after treatment with ciprofloxacin only, indicating an increased number of neutrophilic granulocytes that was consistent with a previous study [33]. We also demonstrated that the CIP–MSN had an insignificant effect on the oxidative stress enzyme activity in the challenged group when compared with the control positive group and the normal ciprofloxacin treatment regime, indicating that this drug-delivery model exhibited no harmful effects on the blood serum. Interestingly, we found that MPO and NO production were reduced in the CIP–MSN-treated group with a lower dose compared with the normal ciprofloxacin treatment regime at 7 and 14 d postchallenge, indicating its important role in abrogating the *S. typhimurium*-induced stimulation of phagocytes and the subsequent health benefit. Tissue injury and inflammation in animals are indicated by elevated secretion of pro-inflammatory cytokines and chemokines by macrophages in the blood serum and liver [34]. Our result showed that *Salmonella typhimurium* challenge significantly increased serum level and liver mRNA expression of pro-inflammatory cytokines and chemokines, which was documented in a previous study [34], and these elevations were abolished by CIP–MSN supplementation at a reduced dose compared to the normal ciprofloxacin treatment regime.

Caspases play a very important role in apoptosis, and their activation takes place upon assembly of an intracellular complex known as inflammasome, which is responsible for the processing and maturation of pro-inflammatory cytokines, such as IL-1β and IL-18 [35,36]. Thus, they act in inflammation and innate immune host defense against microbial pathogens [37]. Previously, *Salmonella typhimurium*w as reported to induce the caspase-dependent death of macrophages upon infection, with the release of pro-inflammatory cytokines that colonize the Peyer’s patches (PPs) and cross the intestinal barrier [38]. These reports prompted us to investigate whether the protective effects of a reduced dose of CIP–MSN supplementation were associated with reduced *S. typhimurium*-induced apoptosis in hepatic cells by analyzing the mRNA expression of pro-apoptotic genes, such as *COX*-2, caspase-3, cytochrome P450, *iNOS*, *Bcl*-2, and *BAX*. As expected, our results revealed increased mRNA levels of pro-apoptotic proteins induced by *Salmonella typhimurium*, indicating infection and increased apoptosis [39]. Accumulating evidence has shown that oxidative stress-related apoptosis is involved in pathogen-infection-induced tissue injury [40]. CIP–MSN administration at half-dose, as compared with the normal ciprofloxacin treatment regime, significantly decreased apoptosis by inhibiting mRNA levels of pro-apoptotic proteins. Consistent with this result, a previous study showed that apoptosis induced by *Salmonella typhimurium* was abolished by therapy supplementation [33].

*S. typhimurium* infection in rats resulted in salmonella-infected phagocytes gaining access to the lymphatics and bloodstream, allowing the bacteria to spread to the liver and the spleen [3]. Previous studies confirmed that the combination of mesoporous silica nanoparticles or silica xerogel and drugs was responsible for effective antibacterial activity when compared with the drug alone, resulting in a more effective clearance of *Salmonella enteric* serovar Typhimurium infection from mouse spleen and liver than the same dose of free drug [14]. In accordance, our results indicated that CIP–MSN at half-dose can boost the clearance rate of *Salmonella enteric* serovar Typhimurium infection in liver and spleen tissues with a significant log reduction of bacterial load than the higher dose of free drug. This counteracted the negative effects of the *S. typhimurium* challenge through a corrected histopathological picture of both the intestine and liver and increased bacterial clearance. Post-*Salmonella typhimurium* infection, the histological pathological architecture of intestinal tissues showed a diffuse inflammatory cell infiltration and complete desquamated epithelial tissues. Meanwhile, with administration of CIP, the severity of intestinal inflammation and liver damage was greatly reduced. Moreover, intestinal and liver tissues that were nearly restored to normal condition were more prominent in the group supplemented with CIP–MSN after *Salmonella typhimurium* infection, indicating its better efficacy in treating infection. Accordingly, treatment with ciprofloxacin and thymol oils against *Shigella flexneri* reduced intestinal infiltration of inflammatory cells in male albino rats and thus reduced the severity of gastric ulcer [41]. Additionally, administration of ciprofloxacin-loaded gold nanoparticles decreased the load of *Enterococcus faecalis* in the liver and kidneys of mice and consequently reduced the severity of tissue damage [42].

## 4. Materials and Methods

### 4.1. Synthesis and Characterization of Mesoporous Silica Nanoparticles (MSNs) and Ciprofloxacin Loading

The mesoporous nanosilica was prepared as previously described at the National Center for Radiation Research and Technology (NCRRT), Atomic Energy Authority, Egypt [43]. The purchased ciprofloxacin (Cipro, Fluka, 98%) was prepared as solution having a concentration of 5 mg/mL, and loaded by incubating with 5 mg of MSN particles for 12 h as previously described [16]. The drug loading capacity was evaluated by measuring the concentration of the free drug in the mixture before and after loading using standard calibration curves obtained by UV–Vis spectroscopy, and calculated by the following equation: loading capacity = weight of ciprofloxacin in MSN particles/weight of MSN particles. In vitro release of ciprofloxacin from MSN particles was determined as previously described [16], in which the dispersed CIP–MSN solution was placed in a dialysis sac, then placed into 50 mL of PBS solution, and shaken at 37 °C. The amount of ciprofloxacin released at different time intervals was evaluated spectrophotometrically at 275 nm (Nanodrop, ND1000, Thermo Scientific, Waltham, MA, USA).

### 4.2. Antibacterial Effect of Ciprofloxacin—Loaded Mesoporous Silica Nanoparticles

The *Salmonellaenterica* serovar Typhimurium ATCC 14028 strain used in this experiment was previously found to be a multivirulent and multidrug-resistant bacterium [44] that has decreased CIP susceptibility, with a minimum inhibitory concentration [9] value of 1 mg/L [7]. Stocks were maintained in 20% (*v*/*v*) glycerol at −80 °C until needed. Bacterial strains were grown on Tryptone Soya Agar (TSA; Oxoid, UK) overnight at 37 °C.

#### 4.2.1. Agar Well Diffusion Assay

The antibacterial activities of CIP–MSN, MSN, and CIP (10 µg/mL concentration) were evaluated against *Salmonella typhimurium,* in which bacterial suspension in sterile saline was prepared to match the optical density of 0.5 MacFarland (1.5 × 108 CFU/mL), and then were grown in Mueller–Hinton (MH) agar (Oxoid Ltd., Hampshire, UK). Wells (8 mm) were cut into each inoculated agar plate, and a 100-µL aliquot of each compound was pipetted into each well. MSN were replaced with sterile water as a negative control for bacterial growth. The plates were incubated at 37 °C for 24 h. After incubation, zones of growth inhibition were measured, and results were expressed as mean ± standard deviation (SD) to determine the antimicrobial potency of the screened compound [45].

#### 4.2.2. Minimum Inhibitory Concentration

The minimum inhibitory concentration was determined using micro broth dilution methods [46]. The concentration of CIP–MSN, MSN, and CIP were serially diluted two-fold, from 1 to 512 μg/mL, and were inoculated with the suspension of standardized inoculum, and then incubated at 37 °C for 24 h. MIC were determined as the lowest concentration that showed no visible growth, while the MBC was determined by culturing 10 μL of each clear well on the Mueller–Hinton agar plates, and the lowest concentration showing a 99.9% reduction in the initial inoculum after overnight incubation was determined as the MBC.

### 4.3. Ciprofloxacin Loaded Mesoporous Silica Nanoparticles Effect on Biofilm Formation

A single colony was inoculated in Mueller–Hinton broth (10^6^ CFU/mL) in microtiter plates with sub-inhibitory concentrations (1/2 × the MIC) of either CIP–MSN, MSN, or CIP, and antibiotic-free medium was used as a negative control. Then, the biofilm formation was performed with the protocol developed previously [47]. The crystal violet was added to measure the extracellular polymeric substances (EPSs) in the biofilm, and the biofilm mass optical density was measured by a microplate ELISA reader (Huma Reader HS, Wiesbaden, Germany) at a wavelength of 590 nm [48]. The XTT cell viability assay kit, according to the manufacturer’s protocol, was employed to measure the viability of cells residing within the matrix [49]. The change in color due to the viability of cells was measured using a microplate reader (Huma Reader HS, German) at a wavelength of 490nm. Experiments were performed in triplicate, and any inferences of nanoparticles in the measurement was deducted from the absorbance imposed by samples, and then the average value was reported with ±SD. The biofilm formation and viable cells were tested in triplicate in independent experiments and interpreted as the ratio of CIP–MSN, MSN, and CIP to the untreated negative control.

### 4.4. Expression of Genes Associated Virulence in Biofilm Culture

qRT-PCR was carried out with the biofilm culture grown in the presence of subinhibitory concentrations of either CIP–MSN, MSN, or CIP, with the untreated negative control as described in a previous section. Then, the bacterial suspension was mixed with RNAprotect Bacteria Reagent (Qiagen, Hilden, Germany) and centrifuged at 5000× *g* for 10 min. RNA extraction was performed using a QIAamp RNeasy Mini kit (Qiagen, Germany, GmbH) according to the manufacturer’s instructions. Genomic DNA was removed from the samples by treatment with 1U DNase I, RNase-free (Thermo Scientific) for 60 min at 37 °C. Real-time PCR amplification reaction was prepared in a final volume 25 µL containing 10 µL of the 2x HERA SYBR^®^ Green RT-qPCR Master Mix (Willowfort, UK), 1 µL of RT Enzyme Mix (20X), 0.5 µL of each primer of 20 pmol concentration, 5 µL of RNase- and DNase-free water, and 3 µL of RNA template. The primer sequences used for the *inv*A (invasion protein) and, *Fim*A (major fimbrial subunit) virulence genes in biofilm culture are shown in Table 1. The 16rRNA gene was used as an internal control for the normalization of the mRNA expression. The PCR products were analyzed using a Step One Real-Time PCR System (Applied Biosystems, California, CA, USA). The comparative Ct method was used to analyze the relative expression of targeted genes, and normalized to the untreated negative control, which was assigned a value of 1 arbitrary unit [50].

### 4.5. Experimental Design, and Oral Challenging with Salmonella typhimurium

This study was conducted in accordance with the regulations approved by the Institutional Animal Care and Use, Faculty of Veterinary Medicine, Zagazig University, and to confirm the freeing of rats from any *Salmonella* spp, bacteriological examinations in accordance with the International Organization for Standardization [51] were conducted on a total of 75 male rats (housed in a standard housing condition). Rats were randomly divided into five different experimental groups with three replicates per each group after one week of adaption.

The experimental treatments were as follows: rats in the first group(negative control, NC) were fed with basal diets without any addition and administered sterile water; rats in the second group (positive control, PC) received a control diet without any addition and were challenged orally with *Salmonella typhimurium* strain (1 × 10^6^ CFU/mL) [16]; those in third and fourth groups received CIP–MSN or MSN orally 12 hr after *Salmonella typhimurium* challenging with 5 mg/kg body weight twice a day for 3 days [16], while the traditional treatment regimen was used in the last group with 10 mg/kg CIP orally twice a day for 3 days. The rats were observed for 14 days postinfection.

### 4.6. Hematological, Biochemical, Oxidative Stress Mediators, and Immunological Measurements

At 7 and 14 d postchallenge, three rats from each treatment were randomly chosen for aseptic collection of blood samples from the tail vein. The collected blood was divided into two equal parts: The first part was collected on heparin as an anticoagulant to determine the blood hematology: red blood cells (RBCs), hemoglobin concentration (Hb), and packed cell volume (PCV) [52]. The second part was immediately centrifuged at 3500 rpm for 15 min, and the serum was used for biochemical biomarkers for liver and kidney injury: alanine aminotransferase (ALT), aspartate aminotransferase (AST),urea, and creatinine, using commercial kits (Span Diagnostic Ltd., Sachin, India). Evaluation of mediators of oxidative stress: nitric oxide (NO) and myeloperoxidase (MPO)were analyzed using commercial kits (Jiancheng Biotechnology Institute, Nanjing, China) [53]. Immunological evaluation of serum chemokines: C-X-C motif chemokine ligand 10 (CXCL10), CC motif chemokine ligand 11(CCL11), pro-inflammatory cytokines; interferon gamma (IFN-γ), interleukin-6 (IL-6), and tumor necrosis factor alpha (TNF-α) were analyzed spectrophotometrically by enzyme-linked immunosorbent assay (ELISA) kits (Cusabio Biotech Co. Ltd., Wuhan, China),and latex-enhanced nephelometry was used for detection of C-reactive protein (CRP) [54].

### 4.7. Quantification of S. typhimurium DNA Copies

Three rats from each experimental group were randomly chosen and slaughtered at 7 and 14 d postchallenge. Liver and spleen tissues were aseptically removed and stored at −80 °C until used; after that, the tissues homogenized, and DNA was extracted according to the manufacturer’s instructions using a QIAamp DNA Stool Mini Kit (Qiagen GmbH, Hilden, Germany), and a NanoDrop2000 spectrophotometer (Thermo Fisher Scientific Inc., Waltham, MA, USA) was used for assessing DNA purity and concentrations. Real-time PCR (RT-PCR) assays were conducted in a Stratagene MX3005P real-time PCR machine for the quantification of DNA copies using generated standard calibration curves from a pure *S. typhimurium* strain, and then interpolating the Ct values of DNA from the liver and spleen samples into the standard curves, and detecting the log_10_ of the CFU numbers 

### 4.8. Pro-Inflammatory Cytokines, and Pro-Apoptotic Gene Expression Analysis by Real-Time PCR

Splenic tissues were also aseptically removed from the same three slaughtered rats, as mentioned in the previous step, and homogenized into RNA later (Sigma, St. Louis, MO, USA) for analyzing the differential gene expressions and immune-related parameters by the RT-qPCR assay. Briefly, total RNA was extracted from the spleen according to the manufacturer’s instructions using Qiagen RNA extraction kits (Cat, No. 74104). Total RNA purity was measured using a NanoDrop_ND-1000 Spectrophotometer (Nano-Drop Technologies, Wilmington, DE, USA). The expression levels of pro-inflammatory cytokines genes: interleukin-6 (*IL-6*),interleukin-1β (*IL-1β*),and tumor necrosis factor-alpha (*TNF-α*), and pro-apoptotic genes:Cyclooxygenase-2 (COX-2), caspase-3, cytochrome-C, inducible nitric oxide synthase (*iNOS*), B-cell lymphoma-2 (*Bcl-2*), and Bcl-2-associated X (*BAX*) as listed in Table 3 were conducted in a Stratagene MX3005P real-time PCR machine using a one-step QuantiTect SYBR Green RT-PCR Kit (Qiagen GmbH, Hilden, Germany) according to the manufacturer’s procedures, and normalized using glyceraldehyde-3-phosphate dehydrogenase (GAPDH) as an internal housekeeping gene. The relative gene expression data were analyzed using the 2−ΔΔCt method and normalized to the untreated negative control which was assigned a value of 1 arbitrary unit [50].

### 4.9. Histopathologic Evaluation

Immediately after the end of the experiment (at 14 dpi), tissue specimens were collected from the liver and intestine tissues, control group, CIP–MSN-, MSN-, and CIP-treated rats (*n* = 3/group). Collected specimens were fixed for 48 h in 10% formalin solution, followed by the routine processing of the specimens, as previously described [44,56]. Thin sections (5 μm) were microtomed and stained with H&E stain and examined under light microscopy. Tissues were blindly examined and evaluated by an experienced pathologist.

### 4.10. Statistical Analysis

The data was analyzed by general linear model (GLM) after confirming the homogeneity among experimental groups using Levene’s test, and normality using Shapiro–Wilk’s test was performed. All data were presented as Mean ± SD. Post-hoc Tukey’s tests were performed to determine if there were significant differences among groups (*p* < 0.05). All statistical analysis and graphical outputs were generated by GraphPad Prism software (Version 8, GraphPad Software Inc.).

## 5. Conclusions

Based on our data, we conclude that, when compared with a normal ciprofloxacin treatment regime, MSN particles loaded with ciprofloxacin at half-dose exhibited a controlled release of the antibiotic that aids in prolonging the antibacterial effect, enhancing in vitro antivirulence efficacy against biofilm formation with reduced levels of adherence and invasion protein expression. Another benefit was its insignificant effect on all blood parameter values, and biochemical biomarkers for tissue injury or inflammation indicated its good biocompatibility and lower cytotoxicity. Additionally, a lower dose of CIP–MSN supplementation has a promising role in reducing inflammatory response and oxidative stress by abolishing the elevated secretion of key mediators of oxidative stress, pro-inflammatory cytokines, and chemokines in the blood serum and liver resulting from *S. typhimurium* infection. Additional evidence supporting the protective effects of a reduced dose of CIP–MSN supplementation established that its association with a reduction in mRNA expression of pro-apoptotic genes resulted in reduced *S. typhimurium*-induced hepatic apoptosis. This counteracted the negative effects of the *S. typhimurium* challenge through a corrected histopathological picture of both the intestine and liver and increased bacterial clearance. Finally, our results indicated that supplementation with CIP–MSN drug-delivery cargo led to a lower antibiotic dose requirement and might be a preventive strategy to alleviate *Salmonella typhimurium*-induced liver injury in humans and animals.

## Figures and Tables

**Table 1:** Zone of inhibition diameter, MIC, and MBC of ciprofloxacin and MSN particles loaded with ciprofloxacin evaluated by agar well diffusion, and broth microdilution methods for *Salmonella typhimurium*.

	Zone of Inhibition (mm)	MIC (mg/L)	MBC (mg/L)
CIP	8.7 ^b^ ± 0.2	1.0	2.0
MSN	3.4 ^c^ ± 0.37	ND	ND
CIP–MSN	40.5 ^a^ ± 0.4	0.03125	0.0625

MIC and MBC are presented in mg/L, and zones of inhibition are presented in mm. CIP–MSN: MSN particles loaded with ciprofloxacin; CIP: ciprofloxacin; MSN: Mesoporous silica particles; MIC: minimum inhibitory concentration; MBC: minimum bactericidal concentration; ND: not determined. Breakpoints for *Salmonella typhimurium* susceptibility profile according to EUCAST guidelines [18]. ^a–c^ Means within the same column carrying different superscripts are significantly different at *p* < 0.05.

**Table 2:** Effects of MSN particles loaded with ciprofloxacin compared with ciprofloxacin on hematological, biochemical, and oxidative stress mediators and the immunological parameters of male rats (7 and 14 days postchallenge with *Salmonella typhimurium*).

	At 7 Days Postinfection							At 14 Days Postinfection						
Groups	NC	PC	CIP	MSN	CIP@ MSN	*p* Value	SEM	NC	PC	CIP	MSN	CIP–MSN	*p* Value	SEM
RBCs	12.5 ^a^	7.96 ^b^	9.17 ^ab^	8.23 ^b^	11.19 ^a^	<0.001	0.09	12.63 ^a^	9.66 ^c^	10.30 ^b^	10.20 ^b^	11.60 ^ab^	0.09	12.63 ^a^
Hb	12.9 ^a^	6.3 ^b^	11.2 ^a^	7.10 ^b^	11.56 ^a^	<0.001	0.07	12.80 ^a^	8.80 ^c^	11.98 ^a^	10.26 ^b^	12.00 ^a^	0.12	12.80 ^a^
PCV	41.4 ^a^	24.5 ^c^	37.43 ^b^	26.60 ^c^	39.5 ^ab^	0.03	0.14	42.30 ^a^	29.69 ^c^	38.6 ^ab^	37.75 ^b^	39.5 ^a^	0.13	42.30 ^a^
	Biochemical biomarkers for tissue injury analysis													
ALT (U/L)	48.3 ^c^	74.7 ^a^	58.7 ^b^	65.63 ^ab^	50.86 ^c^	<0.001	0.21	46.7 ^d^	85.1 ^a^	52.36 ^c^	57.99 ^b^	48.36 ^cd^	0.25	48.3 ^c^
AST (U/L)	25.2 ^b^	42.3 ^a^	27.6 ^b^	26.30 ^b^	25.53 ^b^	<0.001	0.19	24.7 ^c^	48.4 ^a^	25.8 ^c^	34.96 ^b^	23.46 ^c^	0.16	25.2 ^b^
Urea (μmol/L)	32.1 ^c^	52.3 ^a^	38.76 ^b^	50.90 ^a^	35.53 ^bc^	<0.001	0.12	31.6 ^c^	59.8 ^a^	32.6 ^c^	41.90 ^b^	32.1 ^c^	0.14	32.1 ^c^
Creatinine (mg/dL)	1.33 ^b^	2.73 ^a^	1.45 ^b^	1.95 ^ab^	1.41 ^b^	<0.001	0.09	1.34 ^c^	3.07 ^a^	1.38 ^c^	2.35 ^b^	1.33 ^c^	0.08	1.33 ^b^
	Oxidative stress mediators analysis													
NO	153.8 ^d^	532.60 ^a^	246.40 ^b^	480.60	185.36 ^c^	0.027	0.25	150.5 ^e^	559.1 ^a^	202.16 ^d^	140.22 ^b^	177.66 ^c^	0.31	153.8 ^d^
MPO	2.21 ^e^	10.80 ^a^	7.62 ^c^	8.65 ^b^	6.11 ^d^	0.03	0.09	2.16 ^c^	10.08 ^a^	6.76 ^b^		3.57 ^c^	0.10	2.21 ^c^
CRP	1.12 ^e^	54.30 ^a^	24.4 ^c^	50.36 ^b^	16.26 ^d^	0.14	0.14	1.16 ^e^	53.3 ^a^	17.4 ^c^	44.30 ^b^	9.2 ^d^	<0.001	0.08
	Chemokines and pro-inflammatory cytokines analysis													
CXCL10	156.00 ^e^	393.70 ^a^	224.80 ^c^	385.64 ^b^	181.6 ^d^	0.03	0.24	152.6 ^d^	416.3 ^a^	197.9 ^b^	400.02 ^a^	161.83 ^c^	0.02	0.24
CXCL11	108.86 ^e^	238.70 ^a^	161.30 ^c^	220.30 ^b^	130.53 ^d^	0.02	0.29	108.5 ^d^	243.4 ^a^	140.6 ^b^	239.23 ^a^	113.16 ^c^	0.01	0.31
IFN- γ	38.36 ^e^	99.50 ^a^	53.26 ^c^	82.23 ^b^	43.00 ^d^	<0.001	0.11	38.7 ^c^	104.4 ^a^	46.4 ^b^	100.23 ^a^	38.36 ^c^	0.03	0.23
IL-6	16.28 ^c^	47.36 ^a^	47.93 ^a^	45.636 ^a^	34.86 ^b^	<0.001	0.13	15.86 ^c^	47.56 ^a^	34.56 ^b^	49.36 ^a^	16.59 ^c^	<0.001	0.14
TNF-α	11.5 ^c^	23.61 ^a^	16.50 ^b^	20.36 ^a^	16.4 ^b^	<0.001	0.10	11.7 ^c^	21.96 ^a^	15.1 ^b^	22.90 ^a^	13.2 ^c^	<0.001	0.06

NC (negative control): rats received a control diet without any addition; PC (positive control): rats received a control diet without any addition and were orally challenged with *S. typhimurium*; CIP(ciprofloxacin): rats received a control diet, were orally challenged with *S. typhimurium*, and received 10 mg/kg CIP orally twice a day for 3 days; and CIP–MSN (MSN particles loaded with ciprofloxacin): rats received a control diet, were orally challenged with *S. typhimurium*, and received 5 mg/kg CIP orally twice a day for 3 days. RBCs: red blood cells; Hb: hemoglobin concentration; PCV: packed cell volume. ALT: alanine aminotransferase; AST: aspartate aminotransferase; NO: nitric oxide; MPO: myeloperoxidase; CXCL10: C-X-C motif chemokine ligand 10; CCL11:CC motif chemokine ligand 11; IFN-γ: interferon gamma; IL-6: interleukin-6; TNF-α: Tumor Necrosis Factor-alpha; CRP:C-reactive protein. ^a–e^ Means in the same row with different letters are significantly different at (*p* < 0.05).

**Table 3:** Primer sequences utilized for rRT-PCR analysis of targeted gene expression.

Target Gene	Primer Sequence (5′-3′)	Accession No./Reference
iNOS	F-ACCTTCCGGGCAGCCTGTGA R-CAAGGAGGGTGGTGCGGCTG-3′	NM_012611
*COX-2*	F-GCTCAGCC ATACAGCAAATCC R-GGGAGTCGGGCAAT CATCAG	NM_017232
*Caspase-3*	F-GCAGCTAACCTCAGAGAGACATTC R-ACGAGTAAGGTCATTTTTATTCCTGACTT	NM_012922
*Bcl-2*	F-TGCGCTCAGCCCTGTG R-GGTAGCGACGAGAGAAGTCATC	NM_016993
*BAX*	F-CAAGAAGCTGAGCGAGTGTCT R-CAATCATCCTCTGCAGCTCCATATT	NM_017059
*Cytochrome C*	F-TTTGAATTCCTCATTAGTAGCTTTTTTGG R-CCATCCCTACGCATCCTTTAC	NM_012839
*IL-1β*	F-TGACAGACCCCAAAAGATTAAGG R-CTCATCTGGACAGCCCAAGTC	NM_031512.2
*IL-6*	F-CCACCAGGAACGAAAGTCAAC R-TTGCGGAGAGAAACTTCATAGCT	NM_012589.2
*TNF-α*	F-CAGCCGATTTGCCATTTCA R-AGGGCTCTTGATGGCAGAGA	L19123.1
β-actin	F-CGCAGTTGGTTGGAGCAAA R-ACAATCAAAGTCCTCAGCCACAT	V01217.1
*GAPDH*	F-TGCTGGTGCTGAGTATGTCG-3′ R-TTGAGAGCAATGCCAGCC-3′	NM_017008
*invA.*	F-ACAGTGCTCGTTTACGACCTGAAT R-AGACGACTGGTACTGATCGATAAT	[55]
*FimA*	F-TTGCGAGTCTGATGTTTGTCG 62 R-CACGCTCACCGGAGTAGGAT	[55]
*16S rRNA.*	F-AGGCCTTCGGGTTGTAAAGT R-GTTAGCCGGTGCTTCTTCTG	[55]

iNOS: Inducible nitric oxide synthase; COX-2: Cyclo-oxygenase-2; IL: interleukin, *TNF-α*: tumor necrosis factor-alpha, *TGF-β*: transforming growth factor-beta, *COX-2*: cyclooxygenase-2, Bcl-2: B-cell lymphoma-2, BAX: Bcl-2-associated X protein, invA: Invasion protein A, FimA: Major fimbrial subunit.

## Data Availability

Data is contained within the article.
